# Facial tumor in an indigenous child from the Brazilian Amazon

**DOI:** 10.1016/j.jdcr.2023.09.045

**Published:** 2023-11-30

**Authors:** Adryadne da Silva Adolfs, Rosilene Viana de Andrade, Maria Clara da Silva Lima, Henrique Albuquerque, Luciana Mendes dos Santos

**Affiliations:** Heitor Vieira Dourado Tropical Medicine Foundation, Department of Dermatology, Amazon, Brazil

**Keywords:** Amazon, facial tumor, immunohistochemistry, indigenous child

A 5-year-old indigenous child of the Ticuna ethnic group from the town of Benjamin Constant (Amazonas, Brazil) presented an asymptomatic tumor on the face in the previous 5 months without any sign of systemic symptoms. The physical examination revealed an increase in the volume of the right hemiface and the presence of nodules of hardened consistency, adherent to deep planes, slightly brownish in color, and smooth surface with the presence of telangiectasias (Figs 1 and 2). Additionally, there were palpable lymph nodes in the right submandibular chain and one on the left, all of them were approximately 1 cm in size, and some of them had a rigid consistency and were painless on palpation. Incisional biopsy was performed on one of the nodules (Figs 3 to 5).

**Fig 1:**
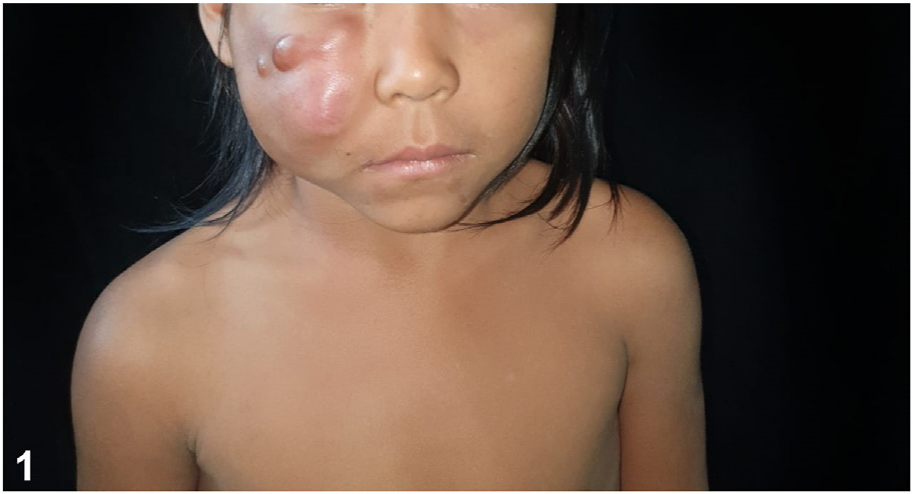

**Fig 2:**
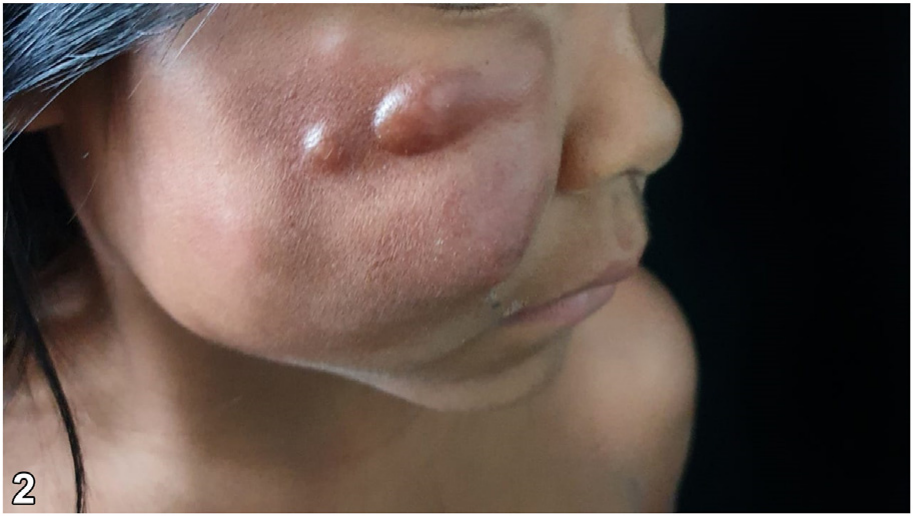

**Fig 3:**
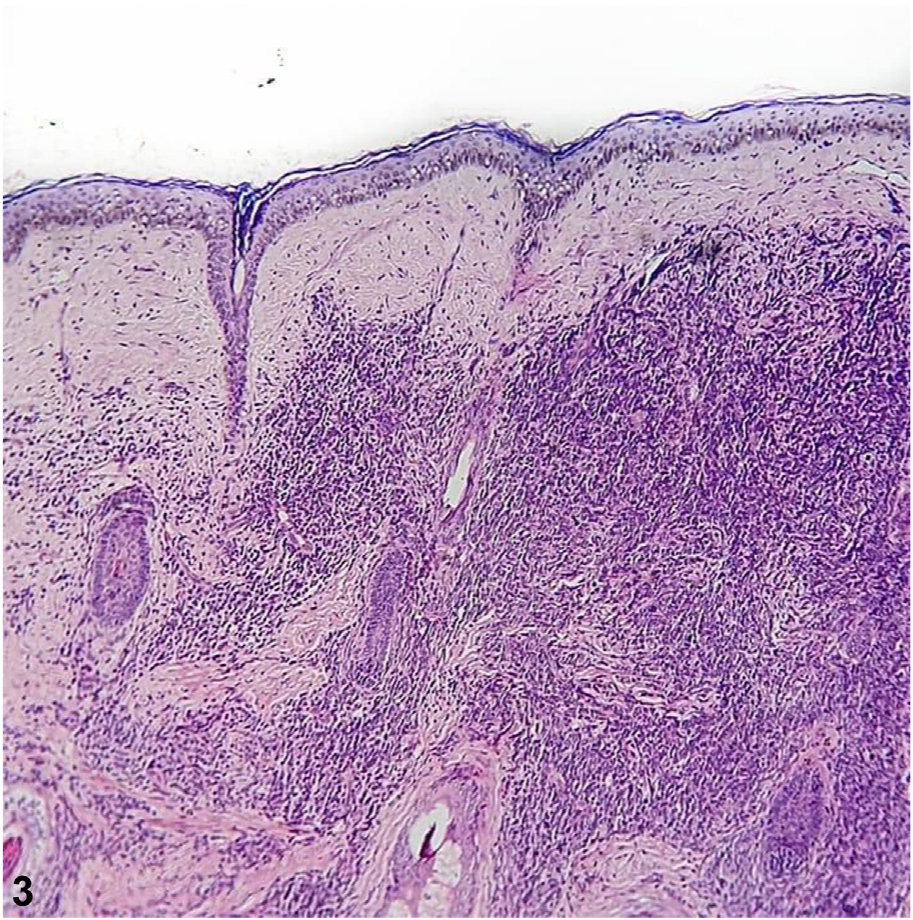

**Fig 4:**
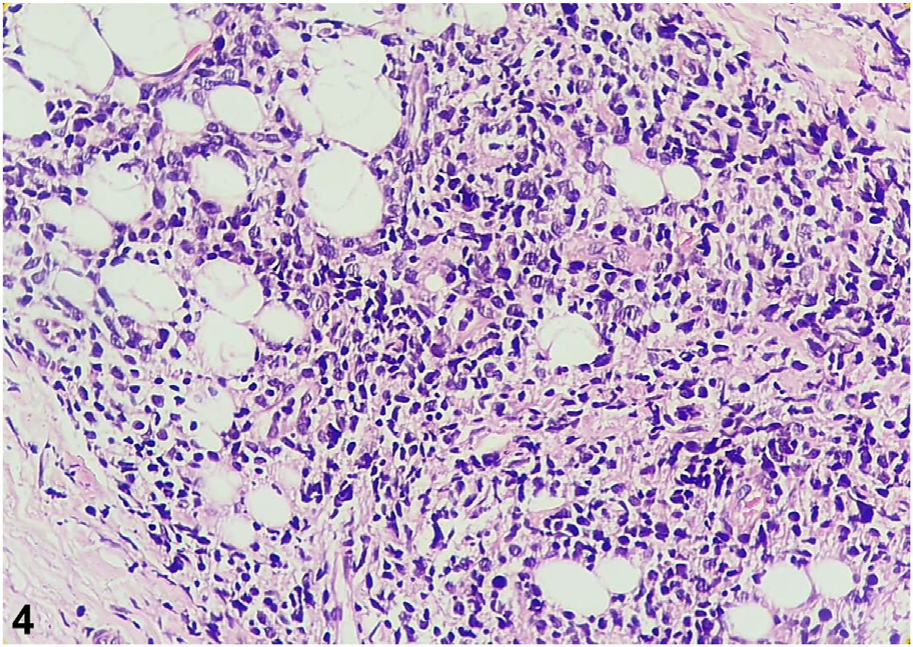

**Fig 5:**
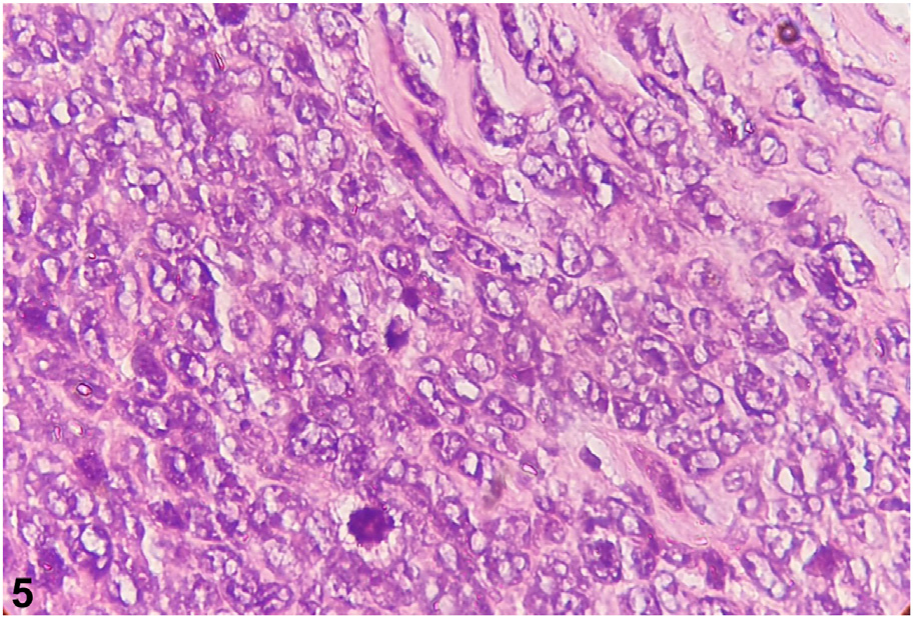

**Question 1: Based on the clinical picture what would be the most likely diagnosis?**

A. Dermatofibrosarcoma protuberans
B. Entomophthoromycosis
C. Ewing sarcoma
D. Lymphoma/leukemia
E. Lobomycosis

**Answers:**

A. Dermatofibrosarcoma protuberans – Incorrect. Dermatofibrosarcoma protuberans is a rare, locally aggressive sarcoma with high recurrence rates and rare metastases. It presents with slow progression and initially appears as a skin tumor of hard consistency, restricted to the skin, and there might be a history of previous trauma. It is common in adults between 2 and 5 decades old. The most frequent location is on the trunk (40%-50%) and proximal extremities (30%-40%).^1^
B. Entomophthoromycosis – Incorrect. Entomophthoromycosis is a subcutaneous mycosis that manifests as a chronic granulomatous infiltration of the nasal mucosa, extending into the subcutaneous tissue and skin of the surrounding face.
C. Ewing sarcoma – Incorrect. Ewing sarcoma is a tumor lesion of aggressive biologic behavior, which is primarily seen on the bone and more rarely appears as a primary tumor of the skin. The clinical presentation is characterized by a superficial tumor mass with an average size of 2 to 3 cm, softened consistency, mobile, and sometimes painful. It affects mainly male individuals under 30 years of age. The most affected sites are the upper and lower extremities, trunk, head, and neck.^2^
D. Lymphoma/leukemia – Correct. Primary cutaneous lymphomas are rare in children and mostly represented by mycosis fungoides and CD30^+^ lymphoproliferative. B- and T-cell non-Hodgkin lymphomas represent about 4% to 6% of malignant neoplasms in children. Lymphoblastic lymphoma (LBL) is defined by a malignant cell neoplasm emerging from the B or T precursor and, is a rare malignant neoplasm usually occurring in the mediastinum of children and adolescents. The B-cell immunophenotype of LBL (B-LBL) accounts for <20% of all cases and may involve extramediastinal areas, such as the skin. It manifests as either primary cutaneous lymphoma or with symptoms secondary to primary extracutaneous disease. Cutaneous lymphoma as erythematous, firm, painless nodules or tumors, between 1 and 6 cm in diameter and growing fast, affecting mainly the head and neck, as shown in this case. The diagnosis must be confirmed by histopathology and immunohistochemistry. The clinical manifestation could be hepatosplenomegaly, lymphadenopathy, fever, musculoskeletal pain, and hematologic abnormalities.^3^, ^4^
E. Lobomycosis – Incorrect. Lobomycosis is a chronic mycosis restricted to the skin and subcutaneous tissue. Although it is endemic in the Brazilian Amazon, the disease is usually frequent in adult males with insidious evolution. Keloidiform lesions are common and occur preferentially on the lower limbs, upper limbs, and ear pinnae.

**Question 2: What are the histopathologic findings suggestive of the disease?**

A. Granulomatous infiltrate of subcutaneous tissue, containing hyaline eosinophilic material, in which hyphae are visualized.
B. Inflammatory, granulomatous, nodular, and diffuse infiltrate, composed of lymphocytes, histiocytes, eosinophils, and plasmocytes, and the presence of a round and birefringent cellular structure.
C. Monomorphic spindle cells with little atypia and mitotic activity, arranged in irregular, multidirectional fascicles (storiform).
D. Normal epidermis, presence of Grenz zone and dense monomorphic infiltrate compound of atypical cells, scanty cytoplasm, dense chromatin, and prominent nucleoli, with the presence of mitotic figures.
E. Small cells with a round, hyperchromatic nucleus. Poorly defined, scanty, light-colored cytoplasm and irregular vacuoles.

**Answers:**

A. Granulomatous infiltrate of subcutaneous tissue, containing hyaline eosinophilic material, in which hyphae are visualized – Incorrect. In entomophthoromycosis, a mononuclear and eosinophilic infiltrate, giant cells, and a wealth of nonseptate hyphae surrounded by eosinophilic material can be seen. Therefore, it is a granulomatous inflammatory infiltrate with the presence of hyphae which confirms the diagnosis of entomophthoromycosis and is not indicative of neoplastic disease.
B. Inflammatory, granulomatous, nodular, and diffuse infiltrate composed of lymphocytes, histiocytes, eosinophils, and plasmocytes, in parallel with the presence of a spherical, birefringent cellular structure – Incorrect. Histopathologic findings are suggestive of lobomycosis, characterized by granulomatous tissue inflammation composed of lymphocytes, randomized histiocytes, eosinophils, and plasma cells. Fungal elements are present in large numbers with a double-contoured, birefringent membrane. Their reproduction by single twinning often leads to the observation of rosary-shaped images. Therefore, birefringent structures are compatible with the yeast-like structures of lobomycosis and do not correspond to neoplasm disease.
C. Monomorphic spindle-shaped cells with poor atypia and mitotic activity, arranged in irregular, multidirectional fascicles (storiform) – Incorrect. This histopathologic feature is characteristic of dermatofibrosarcoma protuberans.^1^ There are no findings such as the Grenz zone, the proliferation of neoplastic lymphocytes in a monomorphic pattern, and frequent mitotic figures, which would indicate LBL.
D. Normal epidermis, presence of Grenz zone and dense monomorphic infiltrate composed of atypical cells, with scant cytoplasm, dense chromatin, and prominent nucleoli, with the presence of mitotic figures – Correct. A histologic study using hematoxylin-eosin staining allows the identification of neoplastic lymphocyte proliferation. In B-LBL the cutaneous histopathologic findings are normal epidermis, a normal band of collagen in the papillary dermis called the Grenz zone, separating the epidermis from the dermal lymphocytic infiltrate, in which the latter consists of a dense monomorphic pattern composed of atypical lymphoid cells, sparse cytoplasm, dotted chromatin, and prominent nucleoli. Mitotic figures are frequent and, in some cases, may form a focal or diffuse stellate sky pattern. Therefore, in the histopathology of the case in question we can observe in hematoxylin-eosin stain at ×100 magnification the normal epidermis, Grenz zone (Fig 3), and a diffuse dermal infiltrate, and at ×400 magnification we can see the infiltrate of atypical cells with irregular nuclei, scanty cytoplasm, and dense chromatin, including replacing the connective tissue (Fig 4) and involving the subcutis (Fig 5), supporting the diagnosis of cutaneous lymphoma, but still requiring immunohistochemistry.^3^, ^4^, ^5^
E. Small cells with a round, hyperchromatic nucleus. Poorly defined, scanty, light-colored cytoplasm and irregular vacuoles – Incorrect. The histopathologic are compatible with Ewing sarcoma, in which small, round uniform cells, hyperchromatic nuclei, and sparse cytoplasm with variable cytoplasmic clearing due to the abundant presence of glycogen are visualized. Mitotic figures are infrequent and aneuploidy is uncommon. On histology, it is observed a similarity between the various small round-cell tumors.^2^ The infiltrate in Ewing sarcoma is not consistent with the infiltrate of atypical lymphocytes with sparse cytoplasm, dotted chromatin, and prominent nucleoli with frequent mitotic figures, besides the Grenz zone is compatible with LBL.

**Question 3: For diagnostic confirmation, immunohistochemistry was requested. Based on this, which markers would be the main ones identified?**

A. CD99 and NKX2
B. Desmin, synaptophysin, MyoD1, and myogenin
C. CD68, CD3, and CD79
D. CD34, hyaluronate, and vimentin
E. CD19, CD10, Ki-67, PAX5, and TdT

**Answers:**

A. CD99 and NKX2 – Incorrect. CD99 is a glycoprotein that has a high level of expression in cells of the hematopoietic system, as well as in cells of Ewing tumors. CD99 is identified in approximately all tumors in the Ewing sarcoma family and, despite not being the most specific, its positivity may contribute to the establishment of the differential diagnosis among small round-cell tumors. NKX2 is an EWS/FLI regulated gene that is required for oncogenic transformation in this tumor, and is a valuable differential marker in the distinction of small round-cell tumors where Ewing sarcoma is considered.^2^^,^^3^
B. Desmin, synaptophysin, MyoD1, and myogenin – Incorrect. These markers are more sensitive and specific for the diagnosis of rhabdomyosarcoma. In this patient, desmin (expressed in the cytoplasm of striated skeletal muscle cells) and synaptophysin (expressed in the cytoplasm of some rhabdomyosarcoma) were also tested, and the results were negative.^3^
C. CD68, CD3, and CD79 – Incorrect. The immunophenotype is compatible with lobomycosis. Immunohistochemistry, in this case, shows positive staining for histiocytes (CD68), T lymphocytes (CD3), and plasma cells (CD79), among other markers.
D. CD34, hyaluronate, and vimentin – Incorrect. Immunophenotype is most commonly seen in dermatofibrosarcoma protuberans.^1^
E. CD19, CD10, Ki-67, PAX5, and TdT – Correct. Immunohistochemistry is crucial for diagnosing and classifying lymphomas, and the antibody panel is streamlined according to histopathologic findings. Their relevance is also for differential diagnosis of small blue pediatric tumors, such as acute myeloid leukemia, Ewing sarcoma, T-cell acute lymphoid leukemia, Burkitt lymphoma, and others. B-LBL/leukemia expresses an immature B cell phenotype and may express CD19, CD79a, TdT, CD10, bbl-2, and CD99 positives, in addition, Ki-67 might be positive due to increased cell proliferation index. Frequently they are CD20 and bcl-6 negative. In the case of this patient, immunohistochemistry was performed, which showed positivity for TdT (lymphoid precursor cell marker) and PAX5 (nuclear marker for B cell), corroborating the diagnosis of B-LBL/leukemia.^3^, ^4^, ^5^

The patient was referred to the reference service in oncohematology tumors, therefor continued investigation with immunohistochemistry confirmed the diagnosis of B-LBL/leukemia, presenting positivity for markers of lymphoid B precursor cell antigens (TdT, marker present in hematopoietic progenitor cells; PAX5, marker present in B lymphocytes in all stages of maturation) and subsequently progressed with pancytopenia. A myelogram was collected, in which small to medium-sized blasts were observed, with a high N/C (Nuclear-to-cytoplasmatic) ratio, basophilic cytoplasm, scanty and agranular, pleomorphic nucleus, and more evident nucleoli. An immunophenotype test was performed that denoted: positive for the markers cCD79a, CD19, CD10, and CD34; negative for the markers cCD3, CD7MPO, CD13, CD33, CD15, CD65, CD20, and cIgM. The patient’s diagnosis was confirmed for B acute lymphoid leukemia and treatment according to BFM 2009 protocol was initiated. Approximately on the 15th day after chemotherapy induction, there was involution of the skin lesion on the face. For cultural/social reasons the patient discontinued the treatment.

## Conflicts of interest

None disclosed.
